# Fitness trade-offs in pest management and intercropping with colour: an evolutionary framework and potential application

**DOI:** 10.1111/eva.12283

**Published:** 2015-08-13

**Authors:** Timothy E Farkas

**Affiliations:** Animal and Plant Sciences, University of SheffieldSheffield, S10 2TN, UK

**Keywords:** agriculture, camouflage, evolutionary adaptation, fitness trade-offs, gene flow, integrated pest management, intercropping, refuge strategy

## Abstract

An important modern goal of plant science research is to develop tools for agriculturalists effective at curbing yield losses to insect herbivores, but resistance evolution continuously threatens the efficacy of pest management strategies. The high-dose/refuge strategy has been employed with some success to curb pest adaptation, and has been shown to be most effective when fitness costs (fitness trade-offs) of resistance are high. Here, I use eco-evolutionary reasoning to demonstrate the general importance of fitness trade-offs for pest control, showing that strong fitness trade-offs mitigate the threat of pest adaptation, even if adaptation were to occur. I argue that novel pest management strategies evoking strong fitness trade-offs are the most likely to persist in the face of unbridled pest adaptation, and offer the manipulation of crop colours as a worked example of one potentially effective strategy against insect herbivores.

## Introduction

A primary concern in agriculture is crop yield loss due to insect herbivores. Many successful methods of pest management have been developed to reduce such losses, dominated in the past century by the use of chemical pesticides applied topically to crops (Ware and Whitacre 2004). More recently, biotechnology has allowed the constitutive expression of pesticides by crop tissues via genetic modification (Christou et al. 2006). Presently, both topical chemical application and genetic modification are widely used pest management practices, especially by large-scale agricultural efforts.

Unfortunately, the efficacy of these practices is gradually eroded due to pest evolution by natural selection. For example, many herbivores have evolved resistance to genetically modified crops expressing crystal protein toxins genetically derived from *Bacillus thuringiensis* (Bt) bacteria (Tabashnik et al. 2008, 2009; Tabashnik et al. 2013; Gassmann et al. 2014; Storer et al. 2010), and insect pests are furthermore known to evolve in response to nonpesticide management practices, such as crop rotation (Krysan et al. 1986; French et al. 2014). Due to the financial cost and difficulty of developing new pest control methods, continuously abandoning good strategies is surely not a sustainable practice. Therefore, a modern aim of pest control is to reduce the abundance and impact of pests while simultaneously limiting pest adaptation to management strategies.

One approach employed to curb adaptation in herbivore pests is the high-dose/refuge strategy (hereafter ‘refuge strategy’). In this approach, farmers use pesticides (or grow Bt crops) on only a portion of their farmed land, allocating the remaining land to a refuge where herbivores are not exposed to pesticides, such that pests in the refuge do not experience natural selection for resistance. The crux of the strategy is that herbivores from refuge populations mate within populations exposed to pesticides and thus introduce susceptible alleles that prevent the otherwise inevitable rise in resistant pest genotypes. Furthermore, using high doses of pesticides makes resistance alleles functionally recessive, such that heterozygotes harbouring both resistant and susceptible alleles experience mortality as severely as susceptible homozygotes. Coupling refuges with high doses is in concept so promising that even governmental agencies have become involved by mandating that farmers create pesticide refuges to prevent regional pesticide resistance evolution that threatens crop yields (Environmental Protection Agency 1998). Recently, the refuge strategy has been shown successful at limiting the evolution of herbivores to Bt crops, demonstrating the practical power of evolutionary theory for agriculture (Carrière et al. 2012; Tabashnik et al. 2013; Siegfried et al. 2014).

**Figure 1:**
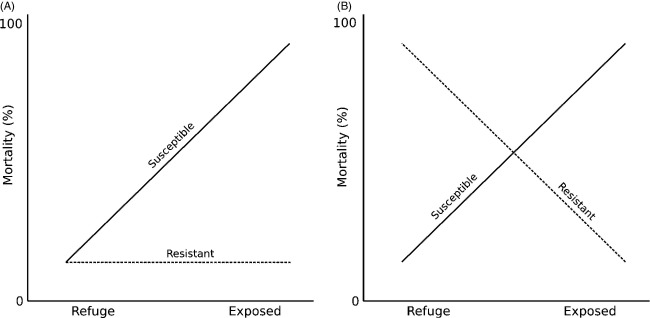
Fitness trade-off topologies. (A) No fitness trade-off. Pests susceptible when exposed to the management strategy experience severe mortality in these crop regions. Resistant genotypes do not experience mortality in the exposed region, but also suffer no fitness cost to resistance, experiencing low mortality in refuges as well. (B) A strong fitness trade-off. Susceptible genotypes experience high mortality in exposed regions and low mortality in refuges. Resistant genotypes experience low mortality in exposed regions, but high mortality in refuges.

An important factor shown theoretically and widely recognized to be important for the efficacy of the refuge strategy is a fitness cost to pesticide resistance (Carrière and Tabashnik 2001; Pittendrigh et al. 2004; Gassmann et al. 2009). A fitness cost to pesticide resistance results in a fitness trade-off, where resistant forms perform better than susceptible forms when pesticides are present, but perform worse when pesticides are absent (Fig.1, Gassmann et al. 2009; Cao et al. 2014). Early modelling efforts demonstrate that the pace of resistance evolution decreases with the strength of a fitness cost to resistance, and that susceptibility can evolve in previously resistant populations if fitness costs are high enough (Carrière and Tabashnik 2001; Pittendrigh et al. 2004). Over the past couple decades, empirical work has accumulated demonstrating the common occurrence but low strength of fitness costs to pesticide resistance (reviewed in Gassmann et al. 2009; Cao et al. 2014), and researchers are actively pursuing methods by which to increase the strength of fitness costs in Bt agrosystems (Pittendrigh et al. 2004; Carrière et al. 2010).

In this study, I use eco-evolutionary reasoning to demonstrate a heretofore underappreciated consequence of fitness trade-offs for pest adaptation in an agricultural setting. In addition to curbing the rate of pest adaptation, strong fitness trade-offs increase overall pest mortality so as to maintain substantial pest management efficacy in the face of unbridled pest adaptation. This reasoning does not apply solely to fitness trade-offs with respect to pesticide resistance by insect herbivores, but is generally applicable to cases of adaptation by any pest to any management strategy. Thus, it is largely intended to offer insight into the development of novel pest management techniques, rather than to improve an understanding of existing methods.

First, I develop a verbal model that demonstrates how fitness trade-offs are an important factor determining the impact of adaptation on pest mortality and crop yield. Second, I discuss assumptions of this model and the consequences of relaxing them, focusing on the possibility of compensatory evolution. Finally, I provide a worked example of a novel pest management strategy to which adaptation by insect herbivores should come at a high cost, thereby demonstrating the use of fitness trade-offs for developing novel management methods. Finally, I offer an analysis of the feasibility of this approach.

## Adaptation and the consequences of fitness trade-offs

Strong fitness trade-offs can neutralize the impact of pest evolution, even if resistance to a management strategy were to evolve. To see how, consider an agricultural landscape where a management technique is implemented through the refuge strategy, and suppose the refuge strategy works well, preventing adaptation by insect herbivores. The ultimate question, however, should be about how much pest mortality (and consequent reduction in yield loss) has been afforded by this strategy, summing across the entire agricultural landscape. The answer to this question largely depends on the topology of trade-offs associated with pest evolution.

**Figure 2:**
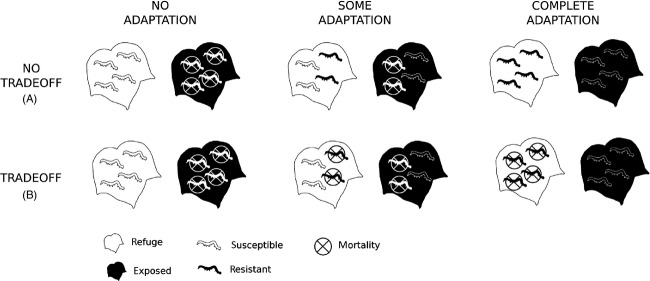
Comparison of the effects of pest evolution on mortality between scenarios for which adaptation has no fitness trade-off (A) versus a strong fitness trade-off (B). Mortality is reduced as adaptation increases when no trade-off is present (A), but evolution has little effect on mortality when there is a strong fitness trade-off (B).

Figure1 shows two out of an infinite set of trade-off topologies for a system with two habitats (exposed versus refuge) and two pest genotypes (resistant versus susceptible). Both topologies are idealized, showing complete versus zero mortality for didactic purposes, and in nature might not be as extreme. In any case, Fig.1A is an example of a topology showing no trade-off. Susceptible (wild type) individuals experience high mortality in regions exposed to a management strategy, but experience low mortality in refuges. Resistant individuals, on the other hand, experience low mortality in both the exposed region and the refuge. Assuming (i) mortalities are nearly 0% or 100%, (ii) half the landscape is planted as refuge, and (iii) panmixia throughout the landscape, if the number of individuals evolving resistance is negligible (i.e., the refuge strategy worked), we predict 50% total mortality summing across the refuge and exposed region: all pests survive in the refuge, and no pests survive in the exposed region. However, pest evolution seriously threatens the efficacy of the management strategy. If 50% of the pest population is resistant, we predict a 25% mortality rate among all pests, and if 100% of the population is resistant, mortality is reduced to zero (Fig.2A). The long-term prospect of a management strategy under these conditions is entirely contingent upon a lack of resistance evolution.

Alternatively, consider the same agricultural scenario, but for which there is a strong trade-off between resistance and susceptibility (Fig.1B). Under this trade-off topology, susceptible individuals still experience high mortality in exposed regions and low mortality in the refuge, but resistant individuals experience low mortality in the exposed region, and high mortality in the refuge. Indeed, the refuge no longer becomes a ‘refuge’, but rather an alternate exposed region (see Pittendrigh et al. 2004). Assuming fitness trade-offs are not perfectly symmetrical and that phenotypic variation in resistance exists, we can expect that adaptation will indeed occur, but the effect of adaptation on mortality is reduced. Supposing 100% of individuals are susceptible, there will be 50% mortality as in the prior example, because all individuals in the exposed region will die, whereas all in the refuge will survive. Unlike the prior example, however, if resistance evolves to 50% in the population, there will still be only 50% mortality, because the half of susceptible individuals in the exposed region will die, and the half of the resistant individuals in the refuge will die. Most strikingly, if 100% of the pests are of the resistant genotype, there will still be 50% mortality, because all of them living in the refuge will die, whereas all of those living in the exposed region will survive (Fig.2B).

An additional feature of pest management strategies eliciting strong fitness trade-offs is that it is unnecessary to ensure resistance is recessive. Without a fitness trade-off, it is very important that resistance is recessive, i.e., that heterozygotes not express resistance. Otherwise, novel resistance alleles could more easily become established in a population and spread to fixation. On the other hand, with strong fitness trade-offs, it makes little difference whether traits relevant to the pest management strategy are recessive, because the effects of pest phenotype on mortality are more symmetrical between the two crop regions. Neither of the genotypes can be considered the ‘resistant’ genotype, as both experience equal mortality.

In summary, the specific topology of fitness trade-offs has a profound influence on the consequences of pest adaptation. As shown above, if fitness trade-offs are weak, then pest adaptation can substantially reduce mortality and render a pest management strategy ineffective. However, if fitness trade-offs are strong, overall pest mortality is largely independent of pest adaptation. Although the verbal model presented above should suffice under most circumstances to aid in the understanding and application of these ideas, I have included a formalized mathematical model in the online supplement that is presented in both discrete-trait and quantitative-trait forms to facilitate deeper exploration.

## Compensatory evolution, phenotypic plasticity and linear yield loss

A major assumption of the model presented above is that pests do not undergo compensatory evolution, wherein the organism evolving resistance, and hence experiencing a fitness trade-off, further evolves to overcome the trade-off and restore high fitness. For example, the evolution of antibiotic resistance has been shown to come at a high fitness cost to microbial pathogens, such as HIV and *E. coli,* but novel mutations subsequently restore high fitness, breaking the fitness trade-off (Schrag and Perrot 1996; Schrag et al. 1997; Levin et al. 2000). For a hypothetical example involving insect herbivores, the evolution of nocturnal feeding could subvert the efficacy of biological control employing visually hunting predators. Although compensatory evolution can be problematic for the efficacy of a pest management strategy, I argue that it is equally problematic for strategies invoking weak and strong trade-offs, and thus does not undermine the argument presented above, which compares two scenarios with contrasting trade-off topologies. Nevertheless, it is useful to outline some of the most potentially problematic instances of compensatory evolution.

The evolution of phenotypic plasticity is one general set of compensatory mechanisms that could be particularly problematic for the efficacy of trade-offs to reduce the impact of adaptation, and the model assumes phenotypic plasticity does not evolve. First, there is no habitat choice that would allow pests to move to and feed in crop regions where their genotype has the highest fitness. Thus, at the start of each generation, there is complete panmixia of genotypes with respect to crop regions. Violation of this assumption in either weak or strong trade-off scenarios would alter model outcomes, depending on the degree to which habitat choice causes covariation in pest genotype and crop region (Zhang et al. 2004; Gore et al. 2005; Binning et al. 2014). The consequence of habitat choice in this context is that trade-offs will be flattened, reducing overall mortality. Second, morphological or physiological plasticity, whereby resistance phenotype becomes uncoupled from genotype and is to some degree determined by the crop region, would similarly undermine the efficacy of pest control. For example, if there evolved a genotype for which the expression of pesticide resistance were determined by pesticide exposure, genotypes expressing such plasticity would likely rise to fixation in the entire population of herbivores, and flatten fitness trade-offs.

Although the verbal model outlined above does not explicitly refer to crop yield, for it to be useful, there must be some assumption linking pest mortality to yield losses. For simplicity, I have assumed (i) that yield loss is related to tissue removal by herbivores only (i.e. not to diseases vectored by pests) and (ii) a linear relationship between pest mortality and yield loss. However, deviations from a linear relationship are known to exist on account of compensatory growth following herbivory for many crops (Maschinski and Whitham 1989; Dyer et al. 1993; Williams et al. 1995; Rosenheim et al. 1997; Suenaga and Hamamura 2015). Nevertheless, the ability of crops to mount compensatory responses to herbivory is highly contextual (Maschinski and Whitham 1989; Rosenheim and Meisner 2013), and herbivory does in some cases cause yield losses that sometimes show a linear, or at least continuous, increasing form (Cardinale et al. 2003; Maas et al. 2013; Rosenheim and Meisner 2013; Liere et al. 2014). Hence, the quantitative application of the theory presented above will necessarily require adjustment to match the biological idiosyncrasies of particular crops and may not apply to cases where crops completely compensate across a realistic range of herbivory.

Next, I demonstrate how strong fitness costs can be put to use developing novel pest control methods by proposing a novel strategy that I believe could be profitably applied to control the abundance of insect herbivores.

## Colour intercropping: A novel technique with a strong fitness trade-off

Camouflage by insect herbivores has been shown to produce a strong fitness trade-off in heterogeneous environments. Consequently, when herbivores use substrates on which they are poorly camouflaged, their abundance can be reduced by more than 50% due to increased predation from visually hunting predators. Furthermore, the effects of poor camouflage in one species can spill over to affect other herbivores, reducing their abundance and herbivory as well, ultimately reducing rates of herbivory by up to 60% (Farkas et al. 2013).

To exploit this trade-off for the purposes of pest control, the strategy requires the development of crop cultivars that differ in tissue coloration. I suggest that this could be done through differential expression of anthocyanins, which are the source of red, purple and blue plant colours (Crozier et al. 2009). Genetic modification, selective breeding, or developmental induction of a plastic response in the production of anthocyanins (Das et al. 2012) could all be methods by which to create crop cultivars with purple tissues. It might even be possible to express anthocyanins in a tissue-specific manner, for example creating cultivars that have purple leaves but whose fruits are a more desirable colour (Shen and Petolino 2006; Lisch 2013).

For implementation, farmers could plant purple cultivars exclusively, instead of green crops, causing sudden widespread camouflage maladaptation for green-coloured herbivores. This strategy should, for a time, reduce herbivory. The problem here is the same as that encountered with pesticide application: herbivore adaptation. Although green is a common colour for externally feeding herbivores, many externally feeding insect pests are known to harbour variation in body colour, including purple, such as in *Spodoptera* caterpillars (Alford 2011). Thus, we should expect that green herbivores, harbouring even slight amounts of heritable body-colour variation, to adapt via natural selection to the purple substrate, evolving purple body coloration to achieve better camouflage.

To combat herbivore colour evolution, the refuge strategy can be used, whereby green and purple cultivars are planted together. This strategy may have two effects. Firstly, planting both green and purple crop regions might act to constrain the evolution of purple pests, due to a strong fitness trade-off. However, mortality of purple individuals on green crops (and vice versa) will likely not be as high as, say, mortality of susceptible pests on Bt crops, where mortality is often greater than 99% (Carrière et al. 2010), and trade-offs are unlikely to be perfectly symmetrical. Hence, we can expect that both phenotypic variation and natural selection will exist for pest populations, casting doubt that green-crop refuges could constrain the evolution of purple pests. However, as I have argued above, if pest genotypes exhibit a strong fitness trade-off across crop regions, mortality will be held nearly constant across a range of pest adaptation if the landscape is intercropped with contrasting plant colours (Fig.2B). Hence, colour intercropping has the potential to increase pest mortality without chance of being fully obviated by pest adaptation.

## Feasibility and future directions for colour intercropping

Whether colour intercropping can serve as an economically viable pest control strategy will require focused investigation in specific agricultural systems. Preliminary work could be done in systems where purple and green varieties currently exist, and are commonly planted, such as cabbage and lettuce, and could be extended to more economically important crops (soy, cotton, corn) as proof-of-concept research accumulates. However, a preliminary analysis of feasibility is useful here by evaluating current knowledge about how camouflage influences pests, how pests influence yield, and the costs and benefits of developing and using anthocyanin-producing crops.

First, whether colour intercropping could actually reduce herbivory enough to substantially curb yield loss is an open and very important question. Depending on the crop, animal pests threaten to reduce crop yields between 9 and 36% (Oerke 2006), but 100% yield restoration is unlikely with colour intercropping. By coupling published research on herbivory and crop yield with research on the effects of camouflage, we can obtain estimates of yield gain due to colour intercropping (see Data S1). Assuming the abundance of externally feeding insect herbivores is 3-fold higher with good camouflage (Farkas et al. 2013), research on aphid herbivory in alfalfa and soy shows that colour intercropping could increase yield by between 8% (Cardinale et al. 2003) and 24% (Liere et al. 2014), based on the effects of only one herbivore species. Based on reductions of an herbivore community on cacao, including caterpillars, beetles, aphids and grasshoppers, yields could be increased by 14% (Maas et al. 2013). These yield gains due to decreased herbivory are not trivial, but other studies show negligible impacts of herbivory on crop yield, emphasizing the need for focused research in specific agricultural systems.

A second line of analysis requires integrating the costs of developing novel crops and the physiological cost of anthocyanin production with the ecological benefit of anthocyanin presence (Kursar and Coley 1992; Dominy et al. 2002; Queenborough et al. 2013). Again, focused investigation in specific crop systems will provide critical information, but we might expect the scales to be tipped towards a net benefit of colour intercropping for a few reasons. First, many crops already have purple cultivars, make the cost of developing them low, and the historical development of purple cultivars indicates that it should be feasible for most crops with relative ease. Second, the well-documented health benefits of dietary anthocyanins to humans (Crozier et al. 2009; Pojer et al. 2013) may encourage their use in global markets, or support subsidies from governments or other organizations interested in promoting public health. Third, the intercropping of green and purple plants could have an added benefit of making weeds more conspicuous and targetable for control methods, and through trade-off logic might combat the potential for weeds to evolve crop mimicry (Barrett 1983). Last, the employment of colour intercropping is an example of a strategy that could be particularly profitable to organic farmers who cannot use many conventional pesticides, or to any farmers for whom methods based on integrated pest management are desirable.

Taken together, these analyses suggest that colour intercropping might be highly effective at curbing yield losses, but caution that any single pest management method might not serve well as a long-term panacea. Hence, I propose that multiple pest management strategies, each evoking strong fitness trade-offs, could be employed simultaneously to increase overall efficacy. It is my hope that fitness trade-offs will inspire agricultural researchers to develop new pest control techniques focused on insect herbivores and other pests, including weeds and microbial diseases, both of which are known to exhibit rapid evolution to attempts at management (Thrall et al. 2011).

One such trade-off-based approach that could be employed alongside colour intercropping is to use pairs of pesticides that drive the evolution of mutual negative cross-resistance. Negative cross-resistance occurs when evolved resistance to one pesticide confers sensitivity to another (Peiris and Hemingway 1990; Pittendrigh et al. 2004); hence, *mutual* negative cross-resistance occurs when the evolution of resistance to either of two pesticides results in sensitivity to the other. The use of negative cross-resistance in the context of the refuge strategy has been discussed before due to its potential to curb resistance evolution (Pittendrigh et al. 2004), but mutual negative cross-resistance would ultimately result in the production of a strong trade-off to resistance evolution, and could reduce the ability of pest evolution to rescue populations from mortality. Future research could pursue pesticide pairs that exhibit mutual negative cross-resistance and that might be employed in tandem to elicit strong trade-offs to adaptation.

## Data archiving

There are no data associated with this manuscript.

## Supporting Information

**Data S1.** Quantitative models and supplementary methods.
