# Supplementary material for: Fitness trade-offs in pest management and intercropping with colour: an evolutionary framework and potential application
Source: Evol Appl. 2015 Aug 13;8(9):847–53. doi: 10.1111/eva.12283 (PMC4610382; doi:10.1111/eva.12283)
Supplement: Supplementary file 1 — Data S1. Quantitative models and supplementary methods. [file eva0008-0847-sd1.docx]

**Data S1.** Quantitative models and supplementary methods.

**Estimating yield gains of colour intercropping**

To estimate yield gains due to colour intercropping, I searched the literature for examples with clear data on the relationship between insect herbivore density and crop yield. In each of three recent examples, each with a different crop (Cardinale et al. 2003, Maas et al. 2013, Liere et al. 2014), I determined the slope of the relationship between abundance and yield, and calculated the expected yield loss for a 3-fold increase in herbivore abundance, to match the findings of Farkas et al. (2013). However, Farkas et al. (2013) compared the difference in arthropod abundance between scenarios with 0% and 100% camouflage. In contrast, the theory presented in the current manuscript suggests that 50% camouflage is a sustainable maximum level across the entire landscape. Hence, the comparison here is between 100% and 50% camouflage, so estimates for yield loss were divided in half.

**Formalized models: The demographic cost of fitness tradeoffs**

*Discrete-trait model*

I begin by formally defining a very general, discrete-trait model describing pest mortality in a complex landscape, in which total pest mortality is equal to the sum of mortality for each pest genotype in each region of the landscape that is distinct with respect to crop type or application of a management strategy:

$m= \sum_{i=1}^{G} \sum_{j=1}^{R} c_{ij}g_{i}r_{j}$ Eqn. 1

where *m* is proportional pest mortality, *G* is the number of functionally distinct pest genotypes, *R* is the number of distinct crop regions, *g_i_* is the proportion of the pest population comprised of genotype *i, r_j_* is the proportion of the landscape of crop region *j*, and *c_ij_* is the mortality rate of individuals of genotype *i* in crop region *j*.

To use this model for an understanding of how fitness costs influence the predictions of pest mortality under scenarios demonstrating pest adaptation, we investigate a simple scenario with only two crop regions and two genotypes (*R = G =* 2). For purposes of accessibility, we discus pest genotypes and crop regions in terms of pest adaptation to a pest management strategy with which the refuge strategy is employed, e.g., Bt crops. Here, one region is planted as a “refuge” (*i* = 1), while the other is “exposed” to a pest management strategy (*i* = 2), and the size of these regions is equal (*r_1_ = r_2_* = 0.5). One genotype is susceptible to the management strategy (*j* = 1) and the other is resistant (*j* = 2). Next, we specify two models: one is parameterized to reflect a strong fitness cost of adaptation, and another in which this fitness cost is set to zero. Note that the mortality model is linear, allowing easy interpolation between predictions at these two extremes. In the model with strong fitness costs to adaptation there exists a complete fitness tradeoff (Fig 1B), such that *c_1,2_* = *c_2,1_* = 1 and *c_1,1_* = *c_2,2_* = 0. In the model with no fitness costs there exists mortality in only one region, such that *c_2,1_*= 1, and *c_2,2_* = *c_1,1_* = *c_1,2_* = 0, and thus there is no fitness tradeoff (Fig 1A).

With these two models specified, we turn to a comparison between them in the effect of adaptation. In terms of model parameters in Eqn. 1, this represents varying *g_i_* values across their range, from 0 to 1, where *g_1_* = 1 – *g_2_*. When pest adaptation is non-existent (*g_2_* = 0), the magnitude of fitness costs makes no difference to pest mortality, and the two models make identical predictions, which is of 50% mortality, summing across both refuge and exposed regions of the landscape. However, the high-cost model begins to win out over the no-cost model if pest adaptation reaches non-negligible levels (*g_2_* > 0). Suppose 10% of pests evolve resistance (*g­_2_* = 0.1). The no-cost model predicts 0% mortality in the refuge, 90% mortality in the exposed region, and total mortality is 45% -- a 5% reduction in the efficacy of the management strategy due to pest adaptation. In the high-cost scenario, however, there will similarly be 90% mortality in the exposed region, but also 10% mortality in the refuge, yielding 50% net mortality across the landscape – no difference from a scenario in which resistance evolution had not occurred at all (Fig 2).

As the amount of pest adaptation increases, the difference between high- and low-cost scenarios becomes more extreme. With the no-cost model, when pest adaptation reaches 100% (*g_2_* = 1), total mortality is predicted to be 0%. In stark contrast, when pest adaptation reaches 100% in the high-cost model, there is still 50% mortality (Fig 2). The conclusion is that high-cost scenarios, where complete fitness tradeoffs are exhibited, show identical levels of pest mortality regardless of how much adaptation occurs, whereas pest adaptation erodes the effectiveness of management strategies where fitness costs are absent.

*Quantitative-trait models*

Above I use a discrete-trait model to demonstrate the consequences of fitness tradeoffs, whereby genotypes have a one-to-one correspondence to a polymorphism. I use a discrete-trait model for convenience and will also use one below as I develop an example of how tradeoffs might be exploited to create adaptation-proof pest management strategies. However, most phenotypic variation in nature is quantitative rather than discrete, being controlled by multiple loci of small effect (Rockman 2012), so pest management strategies might often seek to focus on quantitative traits of pests.

Whereas single mortality-rate values can reasonably be attributed to individual genotypes in each crop region (*c_ij_* values above) for discrete models, quantitative trait models open the opportunity for complex and non-linear functions to describe mortality rates for populations living in discrete habitats. Mortality in such a scenario can be expressed as

$m= \sum_{i}^{R} r_{i}\int_{z_{min}}^{z_{max}} f_{i}\left( z \right)g\left( z \right)dz$ Eqn. 2

where *R* is the number of distinct crop regions, *r_i_* is the proportion of the total landscape planted with crop region *i*, *z* is a value for a quantitative trait, *f_i_*(*z*) is a function describing the probability of mortality across values of *z*, and *g*(*z*) is a probability density function describing the frequency distribution for *z*.

The function *f_i_*(z) might take many forms, including uniform (no differential mortality across *z*), linear, and quadratic. Crossing linear functions for different regions (Fig 1B) would represent a strong fitness tradeoff, similar to the scenario described above using discrete traits. Although the situation becomes complicated with complex mortality functions, strong tradeoffs will be found whenever fitness maxima for one crop region correspond to minima for another. For example, if mortality curves are quadratic such that deviation in either direction from an optimum trait value increases mortality, fitness tradeoffs can be strong if the optima are sufficiently offset (Fig 1C). In this scenario, evolution to achieve an optimal phenotype in one crop region comes at a cost of low fitness in the other (Fig 1C). A deeper analysis of the consequences of complex mortality functions is beyond the scope of this paper, but would be a profitable direction for future research.

**References**

Cardinale, B. J., C. T. Harvey, K. Gross, and A. R. Ives. 2003. Biodiversity and biocontrol: Emergent impacts of a multi-enemy assemblage on pest suppression and crop yield in an agroecosystem. Ecology Letters 6:857–865.

Farkas, T. E., T. Mononen, A. A. Comeault, I. Hanski, and P. Nosil. 2013. Evolution of camouflage drives rapid ecological change in an insect community. Current Biology 23:1835–1843.

Liere, H., T. N. Kim, B. P. Werling, T. D. Meehan, D. A. Landis, and C. Gratton. 2014. Trophic cascades in agricultural landscapes: indirect effects of landscape composition on crop yield. Ecological Applications 25:652–661.

Maas, B., Y. Clough, and T. Tscharntke. 2013. Bats and birds increase crop yield in tropical agroforestry landscapes. Ecology Letters 16:1480–1487.

Rockman, M. V. 2012. The QTN program and the alleles that matter for evolution: All that’s gold does not glitter. Evolution 66:1–17.


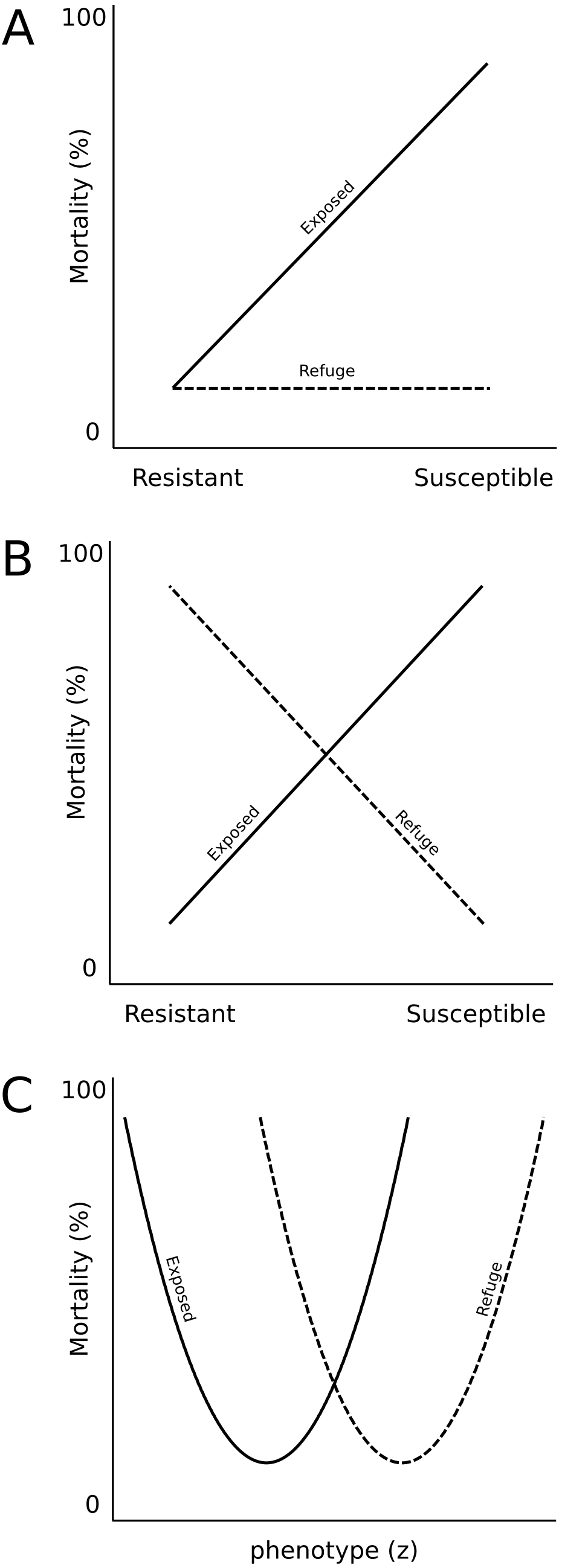


**Figure 1. Fitness tradeoff topologies**. (A, B) Discrete trait mortality. (C) Continuous trait mortality. (A) No fitness tradeoff. Pests susceptible when exposed to the management strategy experience severe mortality in these crop regions. Resistant genotypes do not experience mortality in the exposed region, but also suffer no fitness cost to resistance, experiencing low mortality in refuges as well. (B) A complete fitness tradeoff. Susceptible genotypes experience high mortality in exposed regions and low mortality in refuges. Resistant genotypes experience low mortality in exposed regions, but high mortality in refuges. (C) A complete fitness tradeoff with quadratic mortality functions. The phenotype (z) for which mortality is maximal in one crop region is the phenotype for which mortality is minimal in the other.
